# Pancreatic necrosis as a rare complication of left hemicolectomy—case report

**DOI:** 10.1093/jscr/rjaf1023

**Published:** 2026-01-02

**Authors:** Dulaj Mikołaj, Fiedeń Grzegorz, Klimczak Tomasz, Śmigielski Jacek

**Affiliations:** Regional Hospital in Wieluń, General Surgery Department, 98-300, Poland; Department of General and Oncological Surgery, Cardinal Stefan Wyszynski Hospital in Sieradz, 98-200, Poland; Department of Gastroenterological and Oncological Surgery, Medical University of Lodz, Łódź, 98-200, Poland; Department of General and Oncological Surgery, Cardinal Stefan Wyszynski Hospital in Sieradz, 98-200, Poland

**Keywords:** acute pancreatitis, pancreatic necrosis, sigmoid adenocarcinoma, left hemicolectomy

## Abstract

A 77-year-old female patient was hospitalized due to acute renal failure, during which a colonoscopy was performed because of constipation. The examination revealed an adenocarcinoma of the sigmoid colon. A left hemicolectomy was performed without intraoperative complications. Three days after surgery, the patient reported severe abdominal pain accompanied by nausea and vomiting. Initially, subileus with an anastomotic leak was suspected. A surgical re-exploration of the abdominal cavity was performed. During the procedure, a sealed anastomosis and necrosis of the pancreatic tail were found. Conservative treatment was initiated; however, the patient died 4 days later.

## Introduction

Acute pancreatitis (AP) and/or pancreatic necrosis following colorectal resection is a rarely reported surgical complication. In most cases, AP occurs as a complication of procedures involving the biliary tract, duodenum, or the pancreas itself, as well as in alcohol-related disease. The occurrence of this complication after surgery limited solely to the intraperitoneal cavity remains an unusual phenomenon. According to data available in the PubMed database, only a single well-documented case of AP following right hemicolectomy has been described [1]. It is worth noting that asymptomatic hyperamylasemia is a relatively common finding after abdominal surgeries and is likely related to manipulation within the operative field adjacent to the pancreas. However, the development of full-blown pancreatic necrosis with an unfavorable prognosis is an exceptional clinical situation. A study by Griffith *et al.* demonstrated that hyperamylasemia was observed in 18.7% of patients following colorectal resection, but in most cases, enzyme levels returned to normal within 2 days after the procedure [2].

## Case report

**Figure 1:**
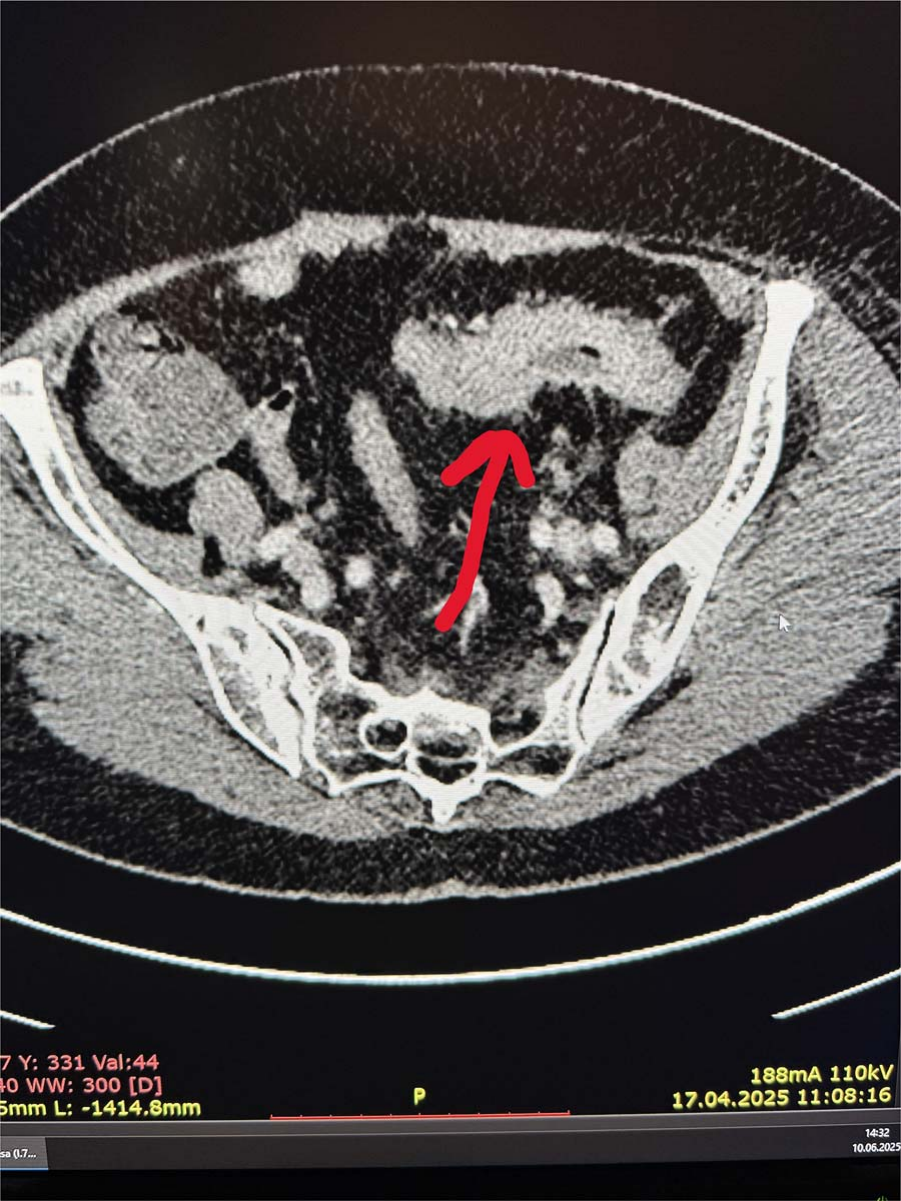
Sigmoid cancer on CT scan.

**Figure 2:**
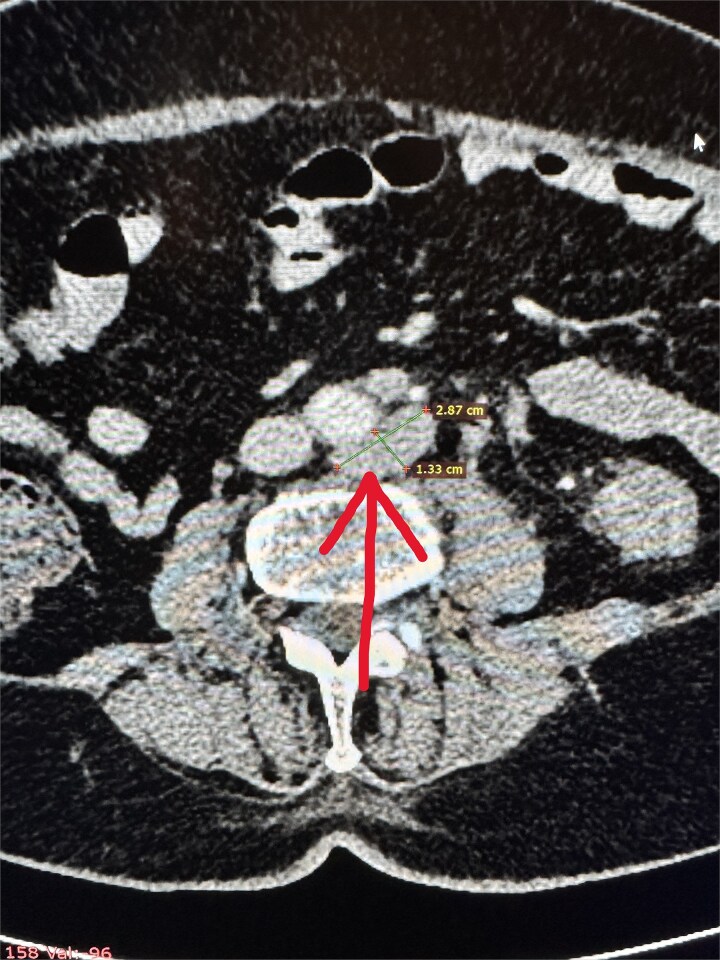
Enlarged para-aortic lymph node.

A 77-year-old female patient was admitted to the nephrology department due to diarrhea leading to dehydration and prerenal kidney failure. Due to accompanying abdominal pain, the diagnostic workup was expanded, and an abdominal computed tomography (CT) scan revealed:

- irregular thickening of the sigmoid colon wall over a length of ~6 cm, with complete obstruction of the lumen in this segment (Fig. 1),
- two cystic lesions on the periphery of the pancreatic tail, up to 7 mm in diameter, and one cystic lesion in the area of the pancreatic head and body (~10 mm),
- numerous enlarged intraperitoneal and para-aortic lymph nodes (Fig. 2).

**Figure 3:**
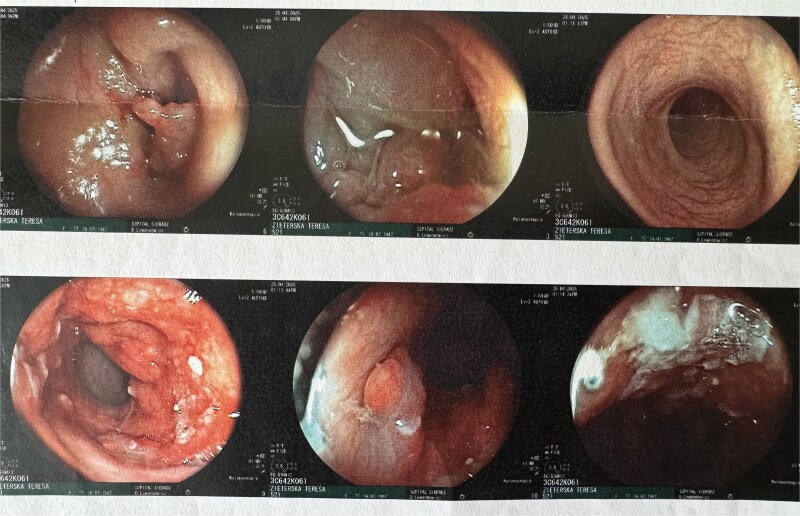
Endoscopic view of colorectal cancer.

A colonoscopy was subsequently performed, which revealed, at ~40 cm from the anal verge, a concentric infiltrative lesion significantly narrowing the intestinal lumen (Fig. 3). An attempt to advance the endoscope resulted in bleeding. Additionally, signs of proctitis were noted. Histopathological examination confirmed a moderately differentiated (G2) adenocarcinoma of the colon.

After stabilization of renal parameters, the patient was qualified for left hemicolectomy. The operation proceeded without intraoperative complications. The bowel was anastomosed using a side-to-end stapled technique.

The first two postoperative days were uneventful. On the third postoperative day, the patient developed abdominal pain, nausea, and vomiting. Blood tests revealed leukocytosis of 43.03 × 10^9^/L, which represented more than a 2-fold increase compared to the previous day, and a C- reaction protein (CRP) level of 21.54 mg/dL. Serum amylase was 285 U/l, and urinary amylase was 10 263 U/l. Due to the lack of clinical improvement and rising inflammatory markers, a decision was made to perform an exploratory laparotomy (Table 1).

**Table 1:** Comparison of laboratory results

Parameter	28.05 (prior to surgery)	02.06 (24 h after surgery)	06.06 (5 days after surgery)
Amylase (IU/l)		285.0	
CRP (mg/dl)	4.35	21.54	7.01
Alat (U/l)	28.0		
Aspat (U/l)	21.0		
Chlorides (mmol/l)	98.8	110.2	112.3
Creatinine (mg/dl)	1.53	0.86	2.22
Urea (mg/dl)	67.0	61.0	204.0
Potassium (mmol/l)	4.5	3.2	3.3
WBC (×10^9^/l)	7.8	48.14	24.77
Sodium (mmol/l)	132.0	144.0	153
GFR	35.0	68.0	22.8

During surgery, the colon anastomosis was inspected and was macroscopically intact. A leak test was also negative. However, extensive adhesions were found around the pancreas, and the tail of the pancreas was enlarged with signs of necrosis. The pancreatic tail formed a conglomerate with the adjacent spleen area and the nearby colon. Intraoperatively, acute necrotizing pancreatitis with Balser’s fat necrosis was diagnosed. In the following days, the patient’s condition progressively deteriorated, with the development of multiple organ failure and sepsis. During the course of treatment, a urinary tract infection (*Enterococcus faecium*) and fulminant kidney failure occurred. Additionally, on the fifth postoperative day, infection of the laparotomy wound (*Klebsiella pneumoniae*) was diagnosed. Despite aggressive management and broad-spectrum targeted antibiotic therapy, the infection progressed to sepsis and the patient died (Table 1).

## Discussion

It is not possible to clearly identify a single mechanism responsible for the development of AP in this case. However, it should be emphasized that such complications are often the result of the accumulation of several unfavorable factors. The possible causes include:

- traction with surgical hooks leading to compression of the pancreatic duct and ischemia of the pancreatic tail,
- mobilization of the splenic flexure of the colon [3],
- potential translocation of intestinal bacteria—Rahman Şenocak *et al.* demonstrated that the number of Gram-negative bacteria in the intestine and pancreatic tissue was significantly higher in rats after colectomy with AP compared to rats with AP alone [4],
- pharmacological factors—there are case reports of propofol-induced AP [5],
- predisposition resulting from small pancreatic cysts detected on CT imaging.

Reports of AP following colorectal surgery remain exceptionally rare. The recent case described by Kay and Gallagher [1] involved AP and enterocutaneous fistula formation following an extended right hemicolectomy. Similar to our patient, their case demonstrated postoperative deterioration, abdominal pain, and markedly elevated pancreatic enzymes. In both reports, intraoperative findings revealed pancreatic necrosis without evidence of an anastomotic leak.

Despite these similarities, several important differences distinguish our case. In the report by Kay and Gallagher [1], extensive mobilization during the right hemicolectomy, right and middle colic vessels ligation were considered potential contributors to pancreatic injury. In contrast, our patient underwent a left hemicolectomy, and no fistula formation was observed. Additionally, preoperative imaging in our case revealed small pancreatic cystic lesions, potentially representing a predisposing factor absent in the previously published case. Taken together, these two cases suggest that although the anatomical approach may differ, pancreatic injury or ischemia associated with peripancreatic traction and splenic flexure mobilization appears to be a shared underlying mechanism.

Based on our experience and data from the literature, we believe that in similar situations it is worth considering the following elements of clinical management:

- Monitoring pancreatic enzyme levels after extensive mobilization of the splenic flexure and large colon resections during the first 3–5 postoperative days, particularly in the case of unclear abdominal symptoms.
- In cases of diagnostic uncertainty, performing abdominal imaging (ultrasound or CT) to differentiate AP.
- During surgery, avoiding excessive retraction with hooks and manipulation around the pancreas, exercising caution when dissecting the colon.
- Including AP in the differential diagnosis of complications after extensive bowel resections.
- In the event of rapid clinical deterioration and high inflammatory markers in a post-colectomy patient, after excluding an anastomotic leak (colonoscopy, dye tests), AP should be considered as a potential cause of clinical worsening.

It appears that the development of necrotizing AP in this patient resulted from a combination of several mechanisms that are not yet fully understood.
